# FCNet: Stereo 3D Object Detection with Feature Correlation Networks

**DOI:** 10.3390/e24081121

**Published:** 2022-08-14

**Authors:** Yingyu Wu, Ziyan Liu, Yunlei Chen, Xuhui Zheng, Qian Zhang, Mo Yang, Guangming Tang

**Affiliations:** 1 College of Big Data and Information Engineering, Guizhou University, Guiyang 550025, China; 2 State Key Laboratory of Public Big Data, Guizhou University, Guiyang 550025, China; 3 Institute of Computing Technology, Chinese Academy of Sciences, Beijing 100190, China

**Keywords:** 3D object detection, deep learning, stereo matching, multi-scale cost–volume, channel similarity, parallel convolutional attention

## Abstract

Deep-learning techniques have significantly improved object detection performance, especially with binocular images in 3D scenarios. To supervise the depth information in stereo 3D object detection, reconstructing the 3D dense depth of LiDAR point clouds causes higher computational costs and lower inference speed. After exploring the intrinsic relationship between the implicit depth information and semantic texture features of the binocular images, we propose an efficient and accurate 3D object detection algorithm, FCNet, in stereo images. First, we construct a multi-scale cost–volume containing implicit depth information using the normalized dot-product by generating multi-scale feature maps from the input stereo images. Secondly, the variant attention model enhances its global and local description, and the sparse region monitors the depth loss deep regression. Thirdly, for balancing the channel information preservation of the re-fused left–right feature maps and computational burden, a reweighting strategy is employed to enhance the feature correlation in merging the last-layer features of binocular images. Extensive experiment results on the challenging KITTI benchmark demonstrate that the proposed algorithm achieves better performance, including a lower computational cost and higher inference speed in 3D object detection.

## 1. Introduction

Object detection is one of the fundamental tasks in computer vision. Three-dimensional object detection in RGB-D images aims to predict the 3D bounding boxes and class labels for each object in applications, such as autonomous driving, mobile robots, and virtual reality augmentation [1,2]. The methods include three categories, i.e., LiDAR-based [3,4,5,6,7,8,9], Monocular-based [10,11,12,13,14,15], and Stereo-based [16,17,18,19]. In the LiDAR-based method, the point clouds generated by expensive LiDAR sensors provide accurate depth information of the 3D space. However, the short operating distance of the LiDAR devices and the sparse point clouds limit its deployment in real scenarios.

In monocular-based methods, predicting objects in 3D space is difficult due to an inherent lack of precise depth cues [10,15,20]. Nonetheless, the fluctuation of inferred depth information influences the suboptimal performance [21]. Thus, stereo cameras offer a reasonable alternative solution, providing denser depth information through left–right photometric alignment and enabling more real-time 3D object detection with considerable accuracy, which provides more data support for controlling the robot, such as cooperative control of manipulators [22] and kinematic control of redundant manipulators [23].

These are the potential applications in low-cost scenarios, such as mobile robots and autonomous driving. The main challenge of stereo-based approaches is the effective acquisition of the implicit depth information, textures, and semantic features in binocular images. Pseudo-LiDAR [20], a commonly used image-based 3D object detection method, converts the depth information into pseudo-LiDAR point clouds and then completes detection using LiDAR-based detection.

The downside to this method is losing accurate semantic information and adding noise when transforming the background. Although designing subsequent point-cloud detection frameworks meet the higher requirements, it requires a vast GPU memory to train the network, which hinders the stereo systems deployment in low-cost applications. Some researchers directly introduced point clouds to improve the detection performance [16,24], while others utilized instance segmentation to focus on the foreground [25,26]. These methods described above essentially transform images into other feature representations; however, they do not thoroughly infer the intrinsic relationship between the implicit depth information, textures, and semantic features. Motivated by the above inspirations, we use the stereo image-based method for 3D object detection with higher accuracy and speed.

**Figure 1:**
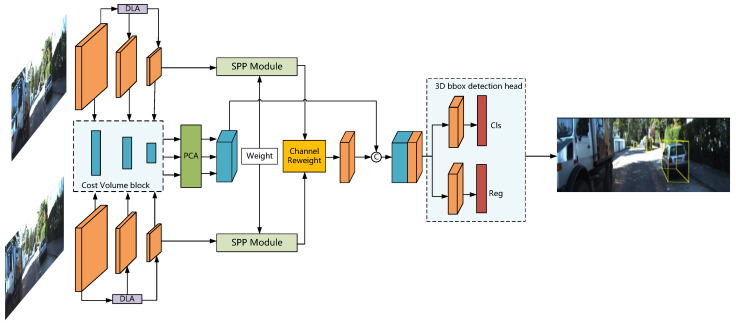
Network inference structure of FCNet. Stereo images are first passed through a simple Siamese network to generate the multi-scale features and then apply a normalized dot-product to construct a multi-scale cost–volume. Finally, we use the parallel attention model and channel similarity reweighting strategy to fuse multi-path features for building the features-based detection heads effectively.

The proposed framework FCNet, as depicted in Figure 1, is trained end-to-end by using stereo image data only. To efficiently correlate sparse depth features in binocular images with semantic and texture features, we introduce the deep-layer aggregation structure for extracting more contextual information. Based on the multi-scale cost–volume with a normalized dot-product, we develop parallel convolutional attention modules to enhance the feature representation and fuse them in top-down processing. Moreover, we formulate the channel similarity reweighting strategy for reducing the redundant features in merging the last-layer features of binocular images. Finally, the combined features generate the base feature of detection heads.

In summary, the main contributions of our work are below:

- A simple and efficient stereo 3D object detection method, FCNet, is proposed. Compared with SOTA approaches, it achieves better performance without LiDAR point clouds and other additional supervision while maintaining an inference time of about 0.1 s per frame.
- After building a multi-scale cost–volume containing implicit depth information, we develop a variant attention module to enhance the global structure representation and local detail description of the multi-scale cost–volume. A region depth loss supervises depth regression.
- A channel reweighting strategy is used to strengthen the feature correlation while integrating the binocular images’ last-layer features by removing the redundant and robust correlation features while retaining the weak correlation features with significant differences. This facilitates the balance between channel information preservation and the computational burden.

## 2. Related Works

### 2.1. Image Depth Estimation

As mentioned previously, 3D object detection using image data is inherently challenging due to the scarcity of reliable depth information. Chen et al. [27] proposed Multi-View 3D Object Detection networks encoded LiDAR point clouds into multi-view feature maps fused with binocular images and predict 3D bounding boxes. Xu et al. [28] applied different sub-networks to process LiDAR point clouds and image data and fused them at the feature level.

Lam et al. [29] adopted a fully convolutional framework. After inputting an RGB image and sparse 3D point clouds to form a sparse depth map and taking the 3D point clouds as depth constraints onto the images, they created an RGB-D image. In addition, some researchers also performed stereo matching to estimate the depth of information. Refs. [30,31] applied the dot-product between binocular feature maps to estimate the disparity distribution.

In PSMNet [32], a spatial pyramid pooling module improves the concatenation-based cost–volumes, and multiple 3D CNN obtains the corresponding depth estimation from rich context information. Unlike the methods of constructing pixel-level cost–volume, TLNet [33] employs 3D anchors to explicitly produce object-level correspondences of the RoI in stereo images, which significantly reduces the depth estimation computational workload. EDNet [34] performs fast stereo estimation using residual attention to aggregate 3D correlation features and 4D concatenation volume on multiple scales and constructs a fast 2D CNN instead of 3D CNN.

In this paper, we take an approach similar to many other stereo 3D object detection algorithms to construct reliable depth features in binocular images, rather than introducing LiDAR point clouds to match the depth information.

### 2.2. 3D Object Detection

**LiDAR-based 3D Object Detection:** In most state-of-the-art 3D object detection methods [3,4,6,8,9,35,36], 3D LiDAR point clouds provide accurate 3D information. In terms of procedures of point clouds, there are two types of streams, i.e., directly operating on the unordered point clouds in 3D [6,8,35,36] and discretizing the locations of point clouds into some voxel grids with a fixed voxel size [3,4,9]. The LiDAR-based methods have high performance, but because of the high-cost LiDAR and huge GPU memory, they are unsuitable for camera-only applications, such as mobile robots and autonomous vehicles.

**Monocular-based 3D Object Detection:** It is challenging to detect 3D objects with a single image for loss of depth information. Mono3D [11] generates 3D bounding boxes from monocular images by utilizing the geometric constraints between 2D and 3D cues, such as semantic and instance segmentation masks. Deep3DBox [21] finishes depth estimation and 3D detection by using angle and scale information. After predicting nine keypoints of a 3D bounding box in image space, RTM3D [12] recovers the location, dimension, and orientation in 3D space by designing a multi-task detection head. Due to the constraints of the target geometric relationship, AutoShape [37] develops more 3D keypoints to improve detection performance.

M3DSSD [13] proposes a two-step feature alignment method to resolve feature mismatching in the context of 2D and 3D Box regression. Unlike the geometric constraint-based methods mentioned above, MonoFENet [14] and DDMP [38] adopted depth estimation to enhance the features of 2D and 3D for accurate 3D localization. MonoGRNet [39] decouples the 3D object detection task into four progressive subtasks, enabling flexible adaptation to fully and weakly supervised learning. Detecting objects in the 3D space with monocular-based methods is difficult due to the shortage of accurate depth information.

**Stereo-Based 3D Object Detection:** Compared with monocular images, stereo images are more appropriate for 3D detection since the disparities between left and right images are conducive to providing more reliable depth information. Despite this, few researchers are concerned about 3D object detection. Pseudo-LiDAR [20] converts the disparity map into 3D point clouds.

Pseudo-LIDAR++ [16] directly obtains a depth map by relying on the depth cost–volume instead of the depth estimation network, effectively reducing computational workload. Disp RCNN [25] predicts disparity only for pixels on objects of interest and generates dense disparity without needing LiDAR point clouds. Similarly, OC-Stereo [26] proposes an object-centric stereo matching method to match differences between foreground and background. By building 4D feature-consistent embedding (FCE) space as the intermediate representation of 3D space, RTS3D [40] improves the detection accuracy without dense supervision information.

Moreover, Stereo-RCNN [17], as a classic image-only 3D detection network, directly applies the existing 2D object detection network to recover a coarse 3D bounding box using left and right RoIs. YOLOStereo3D [18] takes a stereo 3D detection task as a monocular one to enhance stereo features with faster inference speed. The motivations for our work are PSMNet [32] and Stereo-RCNN [17].

## 3. Methods

In this section, we elaborate on the proposed FCNet network structure and the applied methods in this paper. After introducing the anchors’ preprocessing procedures, we present a parallel convolutional attention (PCA) module to produce a more powerful and robust sparse depth feature from binocular images using global context information. We devise a channel similarity reweighting (CSR) strategy to enhance the left and right association feature. Finally, we deliver the multi-task loss function of FCNet.

### 3.1. Anchors Preprocessing

**Figure 2:**
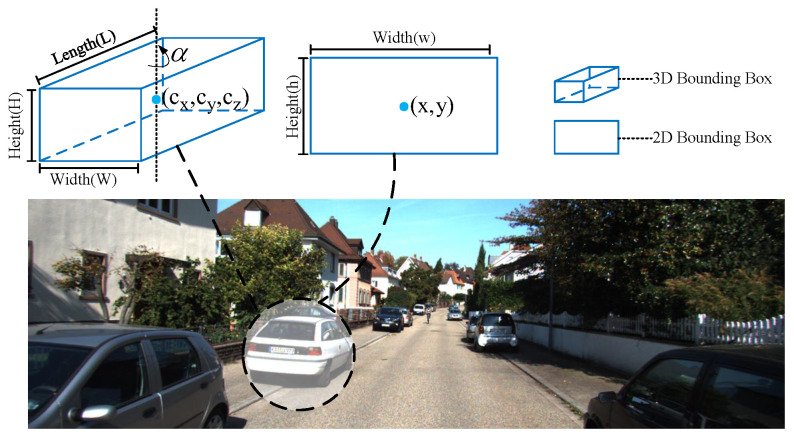
Illustration of the 3D anchor.

**(1) Anchors Definition:** The states of a 3D anchor can be represented by X $=\left\{x,y,w,h,\right.\left.x,c_{y},z,\sin\left(2\alpha \right),\cos\left(2\alpha \right),w_{3d},h_{3d},l_{3d}\right\}$, as shown in Figure 2, where $\left[x,y\right]$ is the center of the left 2D box and $\left[w,h\right]$ is the width and height; $\left[c_{x},c_{y},z\right]$ is the 3D centers of objects, where $\left[c_{x},c_{y}\right]$ is the center of the object projected on the image plane and *z* is the depth; $\left[w_{3d},h_{3d},l_{3d}\right]$ corresponds to the width, height and length of the 3D bounding box, and $\left[\sin(2\alpha ),\cos(2\alpha )\right]$ estimates the observation angle α of objects.

**Figure 3:**
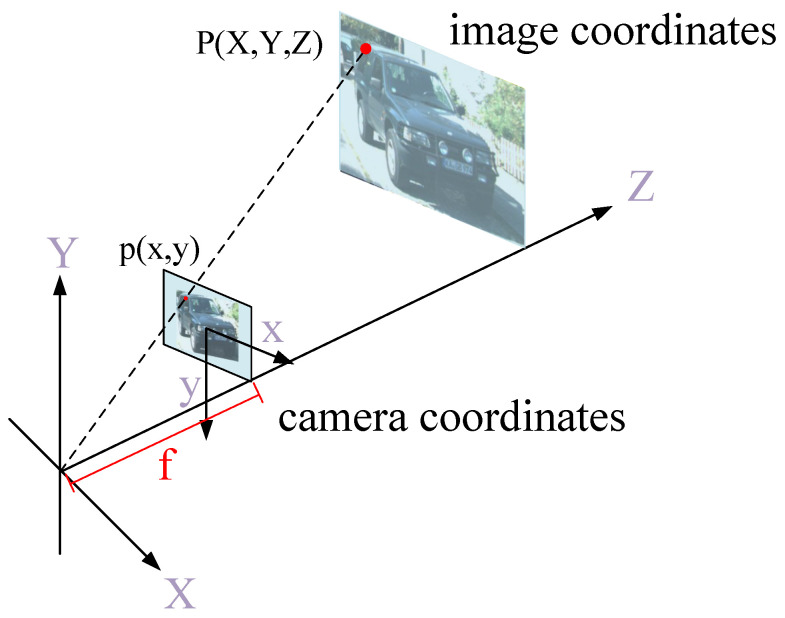
Principles of perspectivegeometry.

**(2) Priors Extraction from label:** As shown in Figure 3, given a 3D point P$=\left(X,Y,Z\right)$ in camera coordinates, its corresponding point $P=\left(x,y\right)$ in image coordinates is given as:

(1) $$x=\frac{X}{Z}f\;y=\frac{Y}{Z}f$$

Suppose the average depth of the object in the 3D space is $\tilde{Z}$, then we can infer the intrinsic relationship between 2D space and 3D space according to Equation (1).

(2) $$Area_{2D}\approx \frac{f^{2}}{{\tilde{Z}}^{3}}Volume_{3D}$$

where $Area_{2D}$ refers to the size of the 2D box, $Volume_{3D}$ is the volume of the object and *f* is the focal length of the camera.

**Figure 4:**
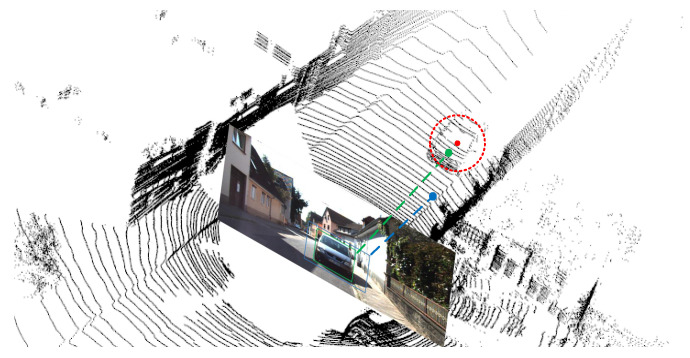
We project anchor boxes with the mean area value *S* of the 2D bounding box into 3D. We filter out anchor boxes with abnormal depth based on the depth value retrieved from the average area during training (the anchor corresponding to the blue point in the figure).

Since the volume of an object in three-dimensional space is relatively constant, we can assume that the anchor depth *Z* is inversely proportional to the object’s size in the image, and we can infer the rough object depth information based on the size of the 2D or 3D bounding box. Thus, we consider each anchor as a distribution with the individual mean and variance of the object proposal in 3D space. After iterating through the training set for utilizing the prior statistical knowledge from the anchor boxes, we estimate the mean *Z* and variance value *S* of the 2D bounding box. In the training period, we convert 2D dense anchor boxes into 3D ones and filter out the anchor boxes with abnormal depth information according to the depth value retrieved from the average area *S*. The schematic diagram illustrates in Figure 4.

### 3.2. Sparse Depth Feature Extraction

Deepening the number of layers is effectively helpful in extracting stereo features. However, it has negative impacts on growing the exponentially computational overhead, which is unsuitable for rapidly changing scenarios, such as autonomous driving.

As shown in Figure 1, we adopt ResNet-34 as the backbone network and apply a deep-layer aggregation structure to give the network better accuracy and fewer parameters. In addition, after using a normalized dot-product to calculate multi-scale cost–volumes, we propose a parallel convolutional attention mechanism to enhance the sparse depth features.

**Figure 5:**
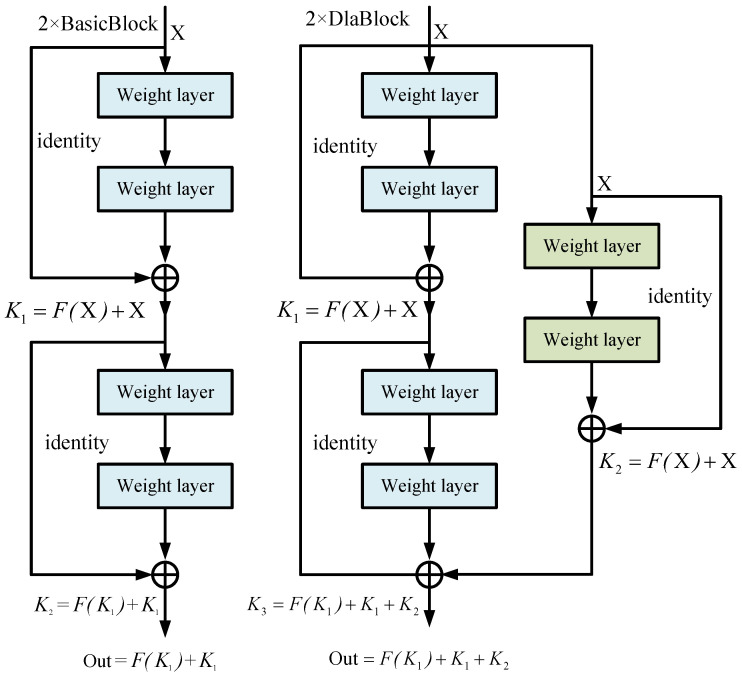
**Deep-layer aggregation structure.** We choose the simplest two conv layers with a kernel size of 3 as the aggregation node to aggregate the features of 2 stacked BasicBlock.

**(1) Deep-Layer Aggregation:** As mentioned above, we propose a deep-layer aggregation structure to extract the richer features. In contrast to directly increasing the number of neurons in each layer, we assemble a residual branch to aggregate features of different layers as shown in Figure 5.

Take the ResNet-6 as the sample, which means 2 × BasicBlocks are stacked. Let the BasicBlock be $F\left(x\right)$. Suppose $x_{in}^{i}$ is the input of the *i*-th BasicBlock, and $K_{i}$ is the output of the *i*-th BasicBlock. In this case, the output $K_{i}$ describes as follows:

(3) $$K_{i}=F\left(x_{in}^{i}\right)+x_{in}^{i}$$

The output of 2 × BasicBlock is written as follows:

(4) $$Out_{Bas}=F\left(K_{1}\right)+K_{1}$$

In the same way, the output of 2 × DlaBlock, which has the deep-layer aggregation, lists as follows:

(5) $$Out_{Dla}=F\left(K_{1}\right)+K_{1}+K_{2}$$

In Equation (5), $K_{2}$ denotes the output of the previous BasicBlock. The network with a deep-layer aggregation structure efficiently reuses the features of different BasicBlocks, which enhances abundant the extracted abundant features due to integrating multiple BasicBlocks.

**(2) Multi-scale Cost Volume:** Instead of supervising the depth information with point clouds, we densely concatenate left feature maps with their corresponding right feature maps across each disparity level to construct a cost–volume block that contains the depth information of the object. As illustrated in Figure 1, the backbone network includes three output branches of different scales, each generating a cost–volume block. To extract more reliable stereo features and exploit more contextual information, we apply the parallel convolutional attention module proposed in the following subsection to merge the cost–volume block of shape sizes $\left[B,max\_disp_{1},H_{1},w_{1}\right]$, $\left[B,max\_disp_{2},H_{2},w_{2}\right]$ and $\left[B,max\_disp_{3},H_{3},w_{3}\right]$ densely. Finally, we use top-down processing to normalize these cost–volume blocks to form a 4D cost–volume with the shape $\left[B,\sum_{i}^{}max\_disp_{i},H_{3},w_{3}\right]$.

**Figure 6:**
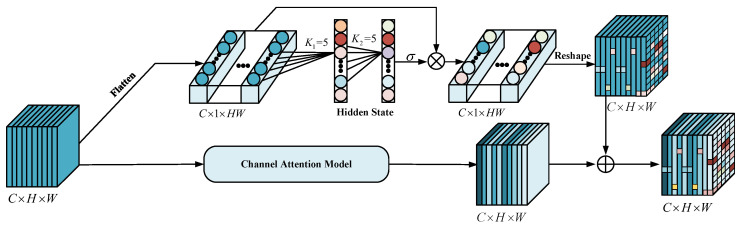
Architectures of the proposed parallel convolutional attention module.

**(3) Parallel Convolutional Attention Module:** Small objects, such as pedestrians have a small proportion of the image’s foreground. It is still difficult to detect small objects accurately for their complex texture features. Thus, a convolutional module, a combination of pixel-level attention and channel attention, is proposed to detect small objects. As shown in Figure 6, given an intermediate feature map $F\in R^{C\times H\times W}$ as input, the module infers, in parallel, a 2D pixel-level attention map $M_{p}\in R^{C\times 1\times HW}$ and a 1D channel attention map $M_{c}\in R^{C\times 1\times 1}$. The overall attention process is summarized as follows:

(6) $$\begin{array}{cc} \; & F^{{}^{\prime }}=M_{c}⊙F\\ \; & F^{{}^{\prime \prime }}=M_{p}⊙R_{C\times 1\times HW}\left(F\right)\\ \; & F=F^{{}^{\prime }}+R_{C\times H\times W}\left(F^{{}^{\prime \prime }}\right) \end{array}$$

where $R_{C\times H\times W}\left(F^{{}^{\prime \prime }}\right)$ represents the size of the attention map $F^{{}^{\prime \prime }}$ into [C×H×W], ⊙ is element-wise multiplication, and $F^{{}^{\prime }}\in R^{C\times H\times W}$ is the final refined output of channel attention. $F^{{}^{\prime \prime }}\in R^{C\times 1\times HW}$ is the final progressive output of pixel-level attention.

To generate the final refined output of channel attention $F^{{}^{\prime }}$ mentioned in Equation (6), We first select the scheme proposed in [41] to create a channel attention map $M_{c}$ and then multiply $M_{c}$ by *F* to get the final output. The entire procedure of channel attention is described as follows:

(7) $$\begin{array}{cc} \; & M_{c}=\sigma (MLP(Avgpool\left(F\right)+MLP\left(Maxpool\left(F\right)\right)\\ \; & F^{{}^{\prime }}=M_{c}⊙F \end{array}$$

where σ and MLP(.) denote the Sigmoid function and a multi-layer perceptron with one hidden layer, respectively. Avgpool(.) and Maxpool(.) represents average-pooling and max-pooling operation separately. Furthermore, the values of pixels in a specific region inextricably link with the depth information of the target, so we use a pixel-level attention module to extract the correlation for neighboring pixels in different regions. Specifically, we flatten an intermediate feature map $F\in R^{C\times H\times W}$ to $F_{1}\in R^{C\times 1\times HW}$, perform two fast 1D convolutions of size 5 to generate the pixel-level attention map $M_{p}\in R^{C\times 1\times HW}$, and multiply $F_{1}$ by $M_{p}$ to obtain the final fine output of pixel-level attention $F^{{}^{\prime \prime }}$. The pixel-level attention is described as follows:

(8) $$\begin{array}{cc} \; & F_{1}=R_{C\times 1\times HW}\left(F\right)\\ \; & M_{p}=\sigma \left(Conv_{1D}\left(Conv_{1D}\left(F_{1}\right)\right)\right)\\ \; & F^{{}^{\prime \prime }}=M_{p}⊙R_{C\times 1\times HW}\left(F\right) \end{array}$$

where σ is the Sigmoid function, and $Conv_{1D}$ is 1D convolution.

**Figure 7:**
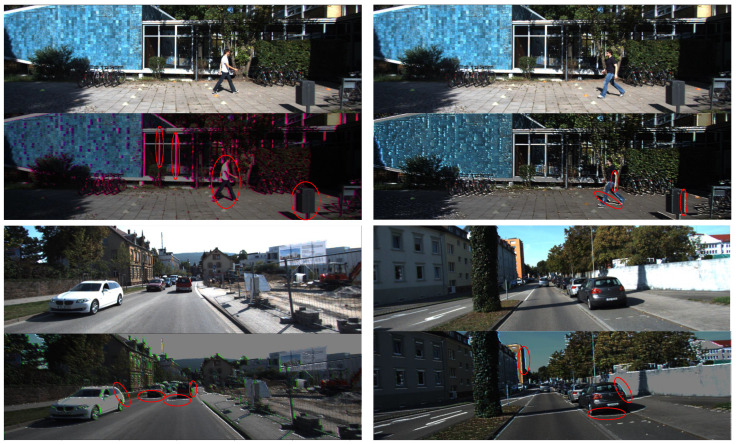
Taking the left images of the stereo pairs as input, the output of the parallel convolutional attention module.

As shown in Figure 7, we apply the pre-trained PCA module to extract features of the camera images. We intuitively notice that the network with the PCA module pays more attention to the pixels at the edge region in images, which highlights by the red ellipse in the in Figure 7.

In addition, as listed in Table 1 and Table 2, the PCA module has superiority in improving performance, especially for pedestrians detection.

### 3.3. Feature Correlation Model

We engineer a correlation-based fusion scheme to utilize the left and right feature maps obtained through the backbone network while reducing feature redundancy.

In Figure 1, taking a pair of left–right feature maps with *C* channels and size H×W as input, we subsequently transfer them into a Siamese Spatial Pyramid Pooling Network to initially find their similarities. Then, we re-fuse the left–right feature maps by utilizing the left–right coherence scores concatenated with spare depth feature maps obtained by stereo matching methods. Finally, we transform them into task-specific fully-connected layers to predict the objectiveness confidence and 3D bounding box offsets.

We choose the absolute value of the Pearson correlation coefficient to calculate the coherence score $S_{i}$ for the *i*th channel in the left–right features maps, which is defined as follows:

(9) $$\begin{array}{cc} S_{i} & =\left|\frac{Cov(F_{i}^{l},F_{i}^{r})}{\sigma_{F_{i}^{l}}\sigma_{F_{i}^{l}}}\right|\\ \; & =\left|\frac{\sum_{j=1}^{H\times W}\left(F_{ij}^{l}-\bar{F_{i}^{l}}\right)\left(F_{ij}^{r}-\bar{F_{i}^{r}}\right)}{\sqrt{\sum_{j=1}^{H\times W}{\left(F_{ij}^{l}-\bar{F_{i}^{l}}\right)}^{2}}\sqrt{\sum_{j=1}^{H\times W}{\left(F_{ij}^{r}-\bar{F_{i}^{r}}\right)}^{2}}}\right| \end{array}$$

where Cov(x,y) is the covariance of *x* and *y*, $F_{i}^{l}$ and $F_{i}^{r}$ are the *i*th pair of the left–right feature maps, $\sigma_{F_{i}^{l}}$ and $\sigma_{F_{i}^{r}}$ are the standard deviations of $F_{i}^{l}$ and $F_{i}^{r}$, $F_{ij}^{l}$ and $F_{ij}^{r}$ are the *j*th element of $F_{i}^{l}$ and $F_{i}^{r}$, $\bar{F_{i}^{l}}$ and $\bar{F_{i}^{r}}$ are the mean value for the *i*th pair of feature maps.

After calculating the coherence scores of all channels in the left and right feature maps, we select channel similarity reweighting (CSR) to enhance the associated representations of the left–right feature maps. Specifically, we still retain the valuable features despite eliminating useless features by setting a threshold to reweight the coherence scores $S_{i}$. The reweighting strategy described as follows:

(10) $$\begin{array}{c} S_{i}^{{}^{\prime }}=\left\{\begin{array}{cc} 0 & x\geq threshod\\ 1-S_{i} & others \end{array}\right. \end{array}$$

**Figure 8:**
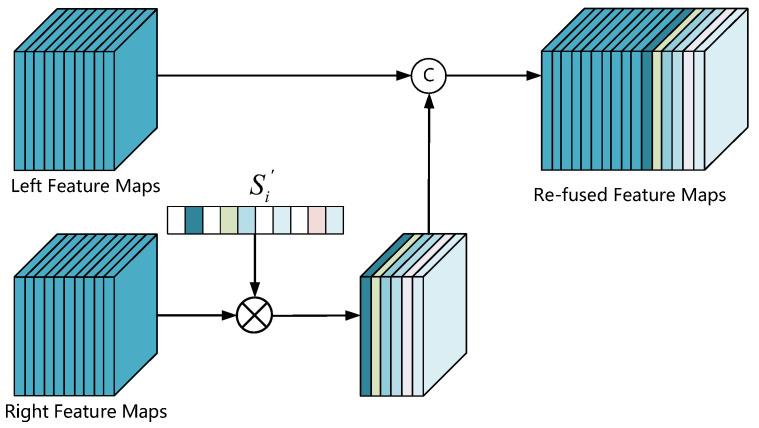
CSR architecture. We first introduce the absolute value of Pearson correlation coefficient to calculate the coherence score $S_{i}$ of each pair of channels, then rescale the coherence score $S_{i}$ based on the reweight threshold, and finally multiply the reweighted coherence scores $S_{i}^{{}^{\prime }}$ with the right feature maps and concatenate it with the left feature maps.

Then, we use the reweighting left–right coherence scores $S_{i}^{{}^{\prime }}$ to re-fuse the left–right feature maps, and the re-fuse process illustrates in Figure 8. By multiplying the *i*th channel of the right feature maps with $S_{i}^{{}^{\prime }}$, we remove the redundant and robust correlation features whose coherence score $S_{i}$ is greater than the threshold value from the right feature maps. After doing this, we retain weak correlation features with significant differences to compensate for the scarcity of features in the left feature maps.

The features with weak correlation are strengthened instead of preserving that of strong correlation because the correlation coefficient of features is larger than the threshold value. The network with the proposed method obtains more discriminative features from stereo images. The positive effects of CSR on 3D object detection shows in Table 1 and Table 2. We verify that the reweighting threshold also affects the network performance, and our experiments’ optimal value threshod is 0.59.

### 3.4. Loss Function

We train the proposed FCNet using a multi-task loss function. This loss function configures four elements, i.e., a classification loss $L_{cls}$, 2D BBox regression loss $L_{2D}$, 3D BBox regression loss $L_{3D}$, and a sparse depth regression loss $L_{Depth}$. The multi-task loss function is defined as follows:

(11) $$Loss=L_{cls}+\lambda_{1}L_{2D}+\lambda_{2}L_{3D}+\lambda_{3}L_{Depth}$$

where $\lambda_{1}$, $\lambda_{2}$, $\lambda_{3}$ are three hyperparameters for balancing different tasks.

The multi-task loss function describes in more detail.

(1) Focal loss function: For object classification, the focal loss [42] deals with the imbalanced classes:

(12) $$L_{cls}=-\alpha_{c}{\left(1-P_{c}\right)}^{\lambda }log\left(P_{c}\right)$$

where $P_{c}$ is the estimated probability of class *c*. $\alpha_{c}$ and λ are the parameters of the focal loss. We use $\alpha_{c}=0.25$ and λ=2 in our training process as in the original paper.

(2) GIoU loss function: As mentioned in Section 3.1, learning 2D bounding box parameters is crucial to filter out anchor boxes with abnormal depth. Therefore, The GIoU [43] loss supervises the regression of the 2D bounding box:

(13) $$L_{2D}=1-\frac{\left|B^{p}\cap B^{g}\right|}{\left|B^{p}\cup B^{g}\right|}+\frac{\left|B^{c}-B^{p}\cup B^{g}\right|}{\left|B^{c}\right|}$$

where $B^{p}$ is the predicted 2D BBox, $B^{g}$ is the ground-truth 2D BBox and $B^{c}$ is the smallest enclosing BBox of $B^{p}$ and $B^{g}$.

(3) Smooth *L*1 loss function: For the 3D BBox regression task, the smooth *L*1 loss function similar to [44] is defined as follows:

(14) $$\begin{array}{cc} \; & L_{3D}=\sum_{d\in P_{3d}}SmooothL1\left(▵d\right)\\ \; & P_{3d}=\left\{x,y,w,h,c_{x},c_{y},z,2\alpha ,2\alpha ,w_{3d},h_{3d},l_{3d}\right\} \end{array}$$

where $\left[x,y\right]$ is the center of the left 2D box and $\left[w,h\right]$ is the width and height; $\left[c_{x},c_{y}\right]$ is the center of the object projected on the image plane and *z* is the depth; $\left[w_{3d},h_{3d},l_{3d}\right]$ corresponds to the width, height and length of the 3D bounding box, and α is the yaw rotation around the z-axis.

(4) Sparse depth regression loss function: We feed the multi-scale cost–volume obtained by stereo matching into a decoder to generate the corresponding sparse depth map and introduce a regional depth loss as an auxiliary loss $L_{Depth}$ to regularize the training process. The sparse depth regression loss $L_{Depth}$ is defined as follows:

(15) $$L_{Depth}=\frac{s^{2}}{w\times h}\sum_{x=x_{g}-w/2s}^{x_{g}+w/2s}\sum_{y=y_{g}-h/2s}^{y_{g}+h/2s}{∥d_{xy}-d_{gt}∥}_{2}^{2}$$

where *w*, *h* are the corresponding width and height of the 2D BBox mapped to the sparse depth map, $\left[x_{g},y_{g}\right]$ is the center of the 2D BBox, *s* is the scaling factor to adjust the size of the selected area, $d_{xy}$ is the depth at position *x*, *y*, $d_{gt}$ is the ground truth depth of the 3D centers of objects.

To optimize the multi-task loss function, we use the Adam optimizer with an initial learning rate of $1\times e^{-4}$ and train for 100 epochs.

## 4. Experiments

### 4.1. Datasets and Evaluation Metrics

We evaluate our method on the challenging KITTI object-detection benchmark [45] for 3D object detection. The KITTI dataset composes 7481 images with labels and 7518 images for testing. Thus, we split 7481 training images into 3712 for the training set and 3769 for the validation set following [18]. To fully evaluate the performance of the proposed FCNet, we follow the official settings to conduct experiments using the 3D Average Precision and 2D Average Precision as the main metrics by comparing to state-of-the-art and self-ablation.

We adopt the ResNet-34 pre-trained on ImageNet as the backbone network in our proposed FCNet and train the network with a batch size of 4 on a single Nvidia GeForce 3060 GPU. We resize the input images to 288×1280. The loss weights $\lambda_{1}$, $\lambda_{2}$ and $\lambda_{3}$ in Equation (11) are $\lambda_{1}=0.7$, $\lambda_{2}=0.8$, $\lambda_{3}=0.4$. Term *s* in Equation (15) is set to 8. As with [13], We use 12 anchor scales ranging from 24 to 288 pixels following the power function of $24\times 12^{i/11}$, $i\in \left\{0,1,2,\ldots ,11\right\}$ and aspect ratios of [0.5, 1.0, 2.0] to define a total of 36 anchors for multi-class detection. As implemented in [17], we use 1024 input channels in the final classification and regression layers.

### 4.2. Ablation Studies

To validate the effectiveness of enhancing our model performance, we conduct ablation experiments on the validation set of Chen’s splits. We train the model on the “Car” and “Pedestrian” types. As shown in Table 1 and Table 2, we obtain the experimental results after 50 epochs of training. Each ablation experiment describes below.

**(1) Deep-Layer Aggregation Structure:** The DLA structure essentially expands the width of the network, improving the reuse of feature maps and guiding each layer to extract richer features.

To test the effectiveness of the DLA structure in boosting the network performance, we switch the status of the network structure with the DLA structure branch or not. As shown in Table 1 and Table 2, Compared with the network without DLA structure, the network with DLA structure achieves +1.64%, +8.26%, +7.98%, +10.8%, +8.53%, +17.79% performance improvements for $AP_{3D}$ on the “Easy”, “Moderate”, and “Hard” categories of car and pedestrian.

**(2) Anchor Preprocessing:** The anchor priors merge into the 3D object detection pipeline during training inference. Thanks to the geometric prior knowledge and constraints, the network further enhances the performance of 3D object detection. We conduct two experiments to verify its effectiveness. In the first experiment, we do not use the prior learned knowledge of BBox, such as the average size and mean square error. In the other experiment, we disable prior-based anchor filtering, i.e., anchors with abnormal depth are not filtered out. The results show that anchor priors significantly boost the network performance, and filtering out irrelevant anchors during training is also necessary to accelerate inference and improve the network performance.

**(3) Parallel convolutional Attention Module:** We also have an ablation study on the importance of parallel attention convolutional modules to improve network performance. The experimental results show that the network with the PCA module achieves practical gains in both car and pedestrian categories, especially for pedestrians with complex textures and edge features. In addition, we visualize the influence of this module on features. As shown in Figure 7, we draw the effect diagrams of the PCA module acting on cars and pedestrians, respectively. We observe from the Figure that the module has apparent effects on the extraction of pedestrian texture features and the division of regional pixels and has a particular impact on the edge calibration and pixel area segmentation of the car.

**Figure 9:**
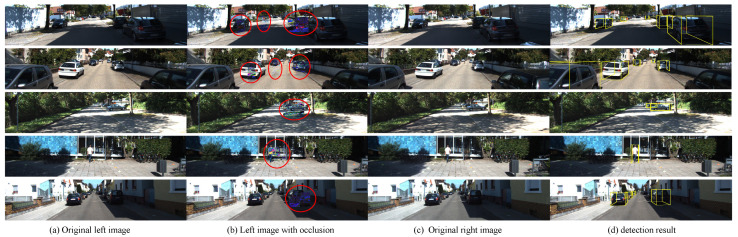
Effectiveness of the reweighting strategy. From left to right: original left images, left images with occlusion, original right images, and detection results.

**(4) Channel Similarity Reweighting:** Reweighting highly similar channels during the fusion between the left and right features significantly reduces noise interference and compensates for channel information loss. To evaluate the effectiveness of the reweighting strategy, we directly fuse the left and right feature maps instead of reweighting-based fusion. According to the results, the reweighting strategy is critical to improving the accuracy of 3D object detection. Furthermore, the reweighting threshold also affects the network performance, and we seek the optimal threshold. To further emphasize the generalization ability and effectiveness of the reweighting strategy, we construct the left image with occlusion by randomly cropping some regions on the original left image to cover the target region (the red circle in Figure 9), and then feed it. The original right image into the detection network, the result is shown in Figure 9. Our proposed network can still effectively detect the target even if interference information exists in the left image.

### 4.3. Qualitative Comparison

**Figure 10:**
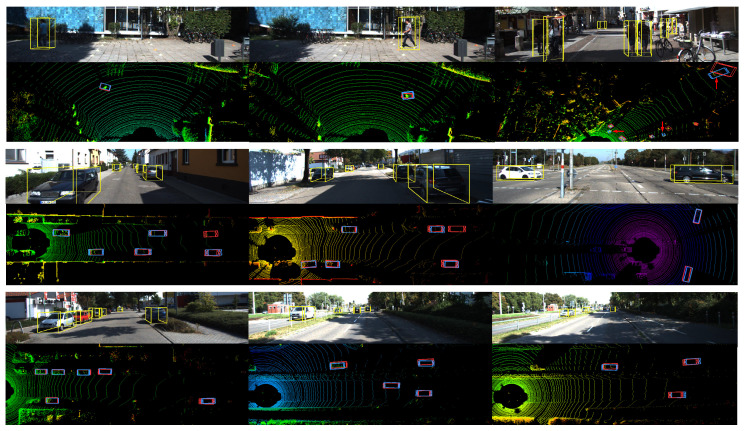
Qualitative results of 3D object detection on KITTI benchmarkt. The RGB images show the detection results on the left images. The LiDAR point clouds are visualized for reference but not used in both training and evaluation.The blue bounding boxes are the 3D prediction from FCNet and the red bounding boxes are the ground truth 3D bounding box.

**Figure 11:**
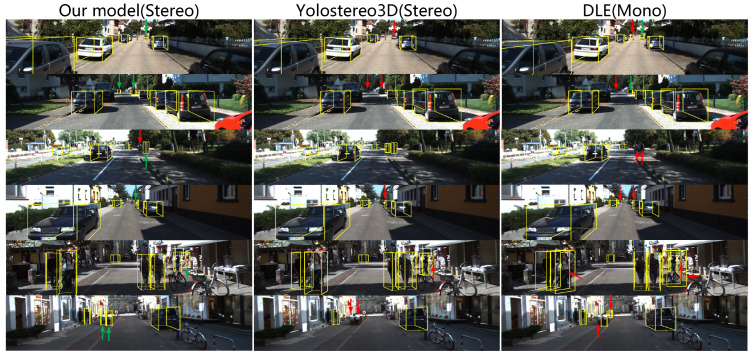
Comparison of our results with other state-of-the-art approaches. Our results are shown in the first column. The remaining second and third columns are results obtained by [18,46], respectively. The red arrows in the figure indicate detected objects or objects with large detection errors, and green arrows indicate validly detected objects.

The qualitative validation results estimated by our FCNet provides in Figure 10. In addition, we also visualize the detection results of other stereo-based 3D object detection frameworks, as shown in Figure 11.

From the results, we observe that, for simple cases of non-occluded objects within a reasonable distance, the most successful predictions of our model are visually consistent with the context. With the proposed model, we correctly detect the pose 3D boxes from other labeled data under incorrectly labeling the original bounding box (e.g., the car pointed by the red arrow in Figure 10). In addition, in some complex cases that seem very challenging in images with lots of nearby or even overlapping 2D boxes, our model can still detect 3D objects correctly. On the other hand, the reasons for several failure detections are that the targets are out of the reachable distance or the presence of severe visual occlusion, which are possible directions for future efforts.

### 4.4. Quantitative Comparison

To further quantitatively evaluate the prediction results of our model, we use the best-trained model in our experiment to calculate the 3D Average Precision ($AP_{3D}$) and 2D Average Precision ($AP_{2D}$) on the KITTI test set and compare the performance of our model with other state-of-the-art methods. The results demonstrate in Table 3 and Table 4. In terms of detection speed, the average inference time of the proposed FCNet is about 0.1s per frame, which is much faster than most other 3D detection networks on the KITTI benchmark (e.g., Pseudo-LiDAR [20], Pseudo-LiDAR++ [16], Disp R-CNN [25] and DSGN [47]).

Regarding $AP_{3D}$, our proposed network FCNet outperforms the most recently proposed methods in 3D detection, significantly outperforming the LiDAR-based method Complex-YOLO by 45.11% on pedestrian detection in the complicated regime. For 2D object detection, we achieve comparable accuracy to state-of-the-art methods. Although FCNet does not compete directly with SOTA methods for 2D detection, its performance is suitable to facilitate the 3D detection task. FCNet has a comparable detection speed to prior art but significantly higher overall precision.

## 5. Conclusions

This paper proposed a novel and practical framework, FCNet, that utilizes the reweighted features and multi-scale cost–volume for 3D object detection. We first applied a deep-layer aggregation structure for widening the backbone to extract richer contextual information and use priors in anchors for depth inference during training to filter out many useless negative samples. Then, we constructed a multi-scale cost–volume and utilized a parallel attention module to enhance the global context information and structural representations in the multi-scale cost–volume fusion stage.

Finally, we used a novel reweighting strategy that balances the channel information preservation of the re-fused left–right feature maps and the computational burden. Comprehensive experiments on the KITTI 3D object-detection benchmark demonstrated that our method achieved better or comparable performance compared to recent SOTA approaches without using LiDAR point clouds and other additional supervision. FCNet also achieved a competitive inference speed with only one GPU with 8 GB GPU memory, which boosts the deployment of 3D object detection on low-cost devices.

In the future, we plan to utilize network pruning to deploy the model on low-cost mobile robots and extend the proposed method to multi-view 3D object detection or video 3D object detection. In addition, we will explore practical methods to reduce the trial and error time for finding the most suitable hyperparameters by using meta-optimization to further improve the system performance.

## Figures and Tables

**Table 1:** Ablation study results of Car on the KITTI validation set.

Methods	${\mathrm{AP}}_{3D}^{0.7}$ of Car (%)		
	Easy	Moderate	Hard
w/o DLA	63.84	38.73	30.19
w/o Anchor Filtering	63.58	39.19	30.14
w/o Bounding Box prior	62.17	37.43	29.84
w/o PCA	63.31	39.42	30.45
w/o CSR	63.94	40.25	30.12
w CSR${}_{0.39}$	64.13	40.87	31.81
w CSR${}_{0.79}$	64.24	41.09	31.97
FCNet	64.89	41.93	32.60

**Table 2:** Ablation study results of Pedestrian on the KITTI validation set.

Methods	${\mathrm{AP}}_{3D}^{0.5}$ of Pedestrians (%)		
	Easy	Moderate	Hard
w/o DLA	29.34	23.07	17.31
w/o Anchor Filtering	31.58	22.89	19.34
w/o Bounding Box prior	29.67	22.51	19.42
w/o PCA	28.94	22.43	17.50
w/o CSR	30.47	23.25	19.17
w CSR${}_{0.39}$	31.71	24.77	19.66
w CSR${}_{0.79}$	31.29	23.79	19.63
FCNet	32.51	25.04	20.39

**Table 3:** 3D detection performance. Comparison of our method to other 3D detection frameworks for Car and Pedestrain categories, evaluted using average precision of 3D bounding boxes ($AP_{3D}$) on KITTI test set. The 3D IoU thresholds are set to 0.7 for car and 0.5 for pedestrian as the same as the official settings.

Methods	Data	Time	${\mathrm{AP}}_{3D}^{0.7}$ of Car (%)			${\mathrm{AP}}_{3D}^{0.5}$ of Pedestrians (%)		
			Easy	Moderate	Hard	Easy	Moderate	Hard
MonoFlex [48]	Mono	0.03 s	19.94	13.89	12.07	9.43	6.31	5.26
MonoRun [15]	Mono	0.07 s	19.65	12.30	10.58	10.88	6.78	5.83
MonoCon [49]	Mono	0.02 s	22.50	16.46	13.95	13.10	8.41	6.94
DLE [46]	Mono	0.06 s	24.23	14.33	10.30	-	-	-
Complexer-YOLO [50]	LiDAR	0.06 s	55.93	47.34	42.60	17.60	13.96	12.70
EGFN [51]	Stereo	0.06 s	65.80	46.39	38.42	14.05	10.27	9.02
Pseudo-LiDAR [20]	Stereo	0.4 s	54.53	34.05	28.25	-	-	-
Pseudo-LiDAR++ [16]	Stereo	0.4 s	61.11	42.43	36.99	-	-	-
Disp R-CNN [25]	Stereo	0.387 s	67.02	43.27	36.43	35.75	25.40	21.79
RT3DStereo [52]	Stereo	0.08 s	29.90	23.28	18.96	3.28	2.45	2.35
Stereo-RCNN [17]	Stereo	0.30 s	47.58	30.23	23.72	-	-	-
OC Stereo [26]	Stereo	0.35 s	55.15	37.60	30.25	24.48	17.58	15.60
Stereo-CenterNet [19]	Stereo	0.04 s	49.94	31.30	25.62	-	-	-
TLNet [33]	Stereo	0.1 s	7.64	4.37	3.74	-	-	-
Yolostereo3D [18]	Stereo	0.1 s	65.68	41.25	30.42	28.49	19.75	16.48
DSGN [47]	Stereo	0.67 s	73.50	52.18	45.14	20.53	15.55	14.15
FCNet(ours)	Stereo	0.1 s	67.83	41.32	31.48	30.15	20.84	18.43

**Table 4:** 2D detection performance. Comparison of our method to other 3D detection frameworks for Car categories, evaluted using average precision of 2D bounding boxes ($AP_{2D}$) on KITTI test set.

Methods	Data	${\mathrm{AP}}_{2D}^{0.7}$ of Car (%)		
		Easy	Moderate	Hard
Faster R-CNN [53]	Mono	87.90	79.11	70.19
MonoGRNet [39]	Mono	88.65	77.94	63.31
RTM3D [12]	Mono	91.82	86.93	77.41
YOLOMono3D [18]	Mono	92.37	79.63	59.69
Mono3D [11]	Mono	90.27	87.86	78.09
MV3D [27]	LiDAR	68.35	54.54	49.16
BirdNet [54]	LiDAR	79.30	57.12	55.16
Stereo-RCNN [17]	Stereo	93.98	85.98	71.25
TLNet [33]	Stereo	76.92	63.53	54.58
Pseudo-LiDAR [20]	Stereo	85.40	67.79	58.50
Yolostereo3D [18]	Stereo	94.81	82.15	62.17
FCNet(ours)	Stereo	94.77	84.53	64.54

## Data Availability

Data are contained with in the article.

## References

[1] Zhou Y., Sun P., Zhang Y., Anguelov D., Gao J., Ouyang T., Guo J., Ngiam J., Vasudevan V. End-to-end multi-view fusion for 3d object detection in lidar point clouds. Proceedings of the Conference on Robot Learning.

[2] Liu Y., Han C., Zhang L., Gao X. (2022). Pedestrian detection with multi-view convolution fusion algorithm. Entropy.

[3] Shi S., Guo C., Jiang L., Wang Z., Shi J., Wang X., Li H. Pv-rcnn: Point-voxel feature set abstraction for 3d object detection. Proceedings of the IEEE/CVF Conference on Computer Vision and Pattern Recognition.

[4] Deng J., Shi S., Li P., Zhou W., Zhang Y., Li H. (2020). Voxel r-cnn: Towards high performance voxel-based 3d object detection. arXiv.

[5] Yang Z., Sun Y., Liu S., Jia J. 3dssd: Point-based 3d single stage object detector. Proceedings of the IEEE/CVF Conference on Computer Vision and Pattern Recognition.

[6] Yang Z., Sun Y., Liu S., Shen X., Jia J. Std: Sparse-to-dense 3d object detector for point cloud. Proceedings of the IEEE/CVF International Conference on Computer Vision.

[7] Qi C.R., Su H., Mo K., Guibas L.J. Pointnet: Deep learning on point sets for 3d classification and segmentation. Proceedings of the IEEE Conference on Computer Vision and Pattern Recognition.

[8] Zhang Y., Huang D., Wang Y. (2020). PC-RGNN: Point Cloud Completion and Graph Neural Network for 3D Object Detection. arXiv.

[9] Zhou Y., Tuzel O. Voxelnet: End-to-end learning for point cloud based 3d object detection. Proceedings of the IEEE Conference on Computer Vision and Pattern Recognition.

[10] Peng L., Liu F., Yan S., He X., Cai D. (2021). Ocm3d: Object-centric monocular 3d object detection. arXiv.

[11] Chen X., Kundu K., Zhang Z., Ma H., Fidler S., Urtasun R. Monocular 3d object detection for autonomous driving. Proceedings of the IEEE Conference on Computer Vision and Pattern Recognition.

[12] Li P., Zhao H., Liu P., Cao F. (2020). Rtm3d: Real-time monocular 3d detection from object keypoints for autonomous driving. Proceedings of the European Conference on Computer Vision.

[13] Luo S., Dai H., Shao L., Ding Y. M3DSSD: Monocular 3D single stage object detector. Proceedings of the IEEE/CVF Conference on Computer Vision and Pattern Recognition.

[14] Bao W., Xu B., Chen Z. (2019). Monofenet: Monocular 3d object detection with feature enhancement networks. IEEE Trans. Image Process..

[15] Chen H., Huang Y., Tian W., Gao Z., Xiong L. Monorun: Monocular 3d object detection by reconstruction and uncertainty propagation. Proceedings of the IEEE/CVF Conference on Computer Vision and Pattern Recognition.

[16] You Y., Wang Y., Chao W.L., Garg D., Pleiss G., Hariharan B., Campbell M., Weinberger K.Q. (2019). Pseudo-lidar++: Accurate depth for 3d object detection in autonomous driving. arXiv.

[17] Li P., Chen X., Shen S. Stereo r-cnn based 3d object detection for autonomous driving. Proceedings of the IEEE/CVF Conference on Computer Vision and Pattern Recognition.

[18] Liu Y., Wang L., Liu M. Yolostereo3d: A step back to 2d for efficient stereo 3d detection. Proceedings of the 2021 IEEE International Conference on Robotics and Automation (ICRA).

[19] Shi Y., Guo Y., Mi Z., Li X. (2022). Stereo CenterNet-based 3D object detection for autonomous driving. Neurocomputing.

[20] Wang Y., Chao W.L., Garg D., Hariharan B., Campbell M., Weinberger K.Q. Pseudo-lidar from visual depth estimation: Bridging the gap in 3d object detection for autonomous driving. Proceedings of the IEEE/CVF Conference on Computer Vision and Pattern Recognition.

[21] Mousavian A., Anguelov D., Flynn J., Kosecka J. 3d bounding box estimation using deep learning and geometry. Proceedings of the IEEE Conference on Computer Vision and Pattern Recognition.

[22] Li S., He J., Li Y., Rafique M.U. (2016). Distributed recurrent neural networks for cooperative control of manipulators: A game-theoretic perspective. IEEE Trans. Neural Netw. Learn. Syst..

[23] Li S., Zhang Y., Jin L. (2016). Kinematic control of redundant manipulators using neural networks. IEEE Trans. Neural Netw. Learn. Syst..

[24] Qian R., Garg D., Wang Y., You Y., Belongie S., Hariharan B., Campbell M., Weinberger K.Q., Chao W.L. End-to-end pseudo-lidar for image-based 3d object detection. Proceedings of the IEEE/CVF Conference on Computer Vision and Pattern Recognition.

[25] Sun J., Chen L., Xie Y., Zhang S., Jiang Q., Zhou X., Bao H. Disp r-cnn: Stereo 3d object detection via shape prior guided instance disparity estimation. Proceedings of the IEEE/CVF Conference on Computer Vision and Pattern Recognition.

[26] Pon A.D., Ku J., Li C., Waslander S.L. Object-centric stereo matching for 3d object detection. Proceedings of the 2020 IEEE International Conference on Robotics and Automation (ICRA).

[27] Chen X., Ma H., Wan J., Li B., Xia T. Multi-view 3d object detection network for autonomous driving. Proceedings of the IEEE Conference on Computer Vision and Pattern Recognition.

[28] Xu D., Anguelov D., Jain A. Pointfusion: Deep sensor fusion for 3d bounding box estimation. Proceedings of the IEEE Conference on Computer Vision and Pattern Recognition.

[29] Huynh L., Nguyen P., Matas J., Rahtu E., Heikkilä J. Boosting monocular depth estimation with lightweight 3d point fusion. Proceedings of the IEEE/CVF International Conference on Computer Vision.

[30] Luo W., Schwing A.G., Urtasun R. Efficient deep learning for stereo matching. Proceedings of the IEEE Conference on Computer Vision and Pattern Recognition.

[31] Zbontar J., LeCun Y. (2016). Stereo matching by training a convolutional neural network to compare image patches. J. Mach. Learn. Res..

[32] Chang J.R., Chen Y.S. Pyramid stereo matching network. Proceedings of the IEEE Conference on Computer Vision and Pattern Recognition.

[33] Qin Z., Wang J., Lu Y. Triangulation learning network: From monocular to stereo 3d object detection. Proceedings of the IEEE/CVF Conference on Computer Vision and Pattern Recognition.

[34] Zhang S., Wang Z., Wang Q., Zhang J., Wei G., Chu X. EDNet: Efficient Disparity Estimation with Cost Volume Combination and Attention-based Spatial Residual. Proceedings of the IEEE/CVF Conference on Computer Vision and Pattern Recognition.

[35] Qi C.R., Yi L., Su H., Guibas L.J. (2017). Pointnet++: Deep hierarchical feature learning on point sets in a metric space. arXiv.

[36] Shi S., Wang Z., Wang X., Li H. (2019). Part-*A*^2^ net: 3d part-aware and aggregation neural network for object detection from point cloud. arXiv.

[37] Liu Z., Zhou D., Lu F., Fang J., Zhang L. Autoshape: Real-time shape-aware monocular 3d object detection. Proceedings of the IEEE/CVF International Conference on Computer Vision.

[38] Wang L., Du L., Ye X., Fu Y., Guo G., Xue X., Feng J., Zhang L. Depth-conditioned dynamic message propagation for monocular 3d object detection. Proceedings of the IEEE/CVF Conference on Computer Vision and Pattern Recognition.

[39] Qin Z., Wang J., Lu Y. (2021). Monogrnet: A general framework for monocular 3d object detection. IEEE Trans. Pattern Anal. Mach. Intell..

[40] Li P., Su S., Zhao H. RTS3D: Real-time Stereo 3D Detection from 4D Feature-Consistency Embedding Space for Autonomous Driving. Proceedings of the AAAI Conference on Artificial Intelligence.

[41] Woo S., Park J., Lee J.Y., Kweon I.S. Cbam: Convolutional block attention module. Proceedings of the European Conference on Computer Vision (ECCV).

[42] Lin T.Y., Goyal P., Girshick R., He K., Dollár P. Focal loss for dense object detection. Proceedings of the IEEE International Conference on Computer Vision.

[43] Rezatofighi H., Tsoi N., Gwak J., Sadeghian A., Reid I., Savarese S. Generalized intersection over union: A metric and a loss for bounding box regression. Proceedings of the IEEE/CVF Conference on Computer Vision and Pattern Recognition.

[44] Yan Y., Mao Y., Li B. (2018). Second: Sparsely embedded convolutional detection. Sensors.

[45] Geiger A., Lenz P., Urtasun R. Are we ready for autonomous driving? The kitti vision benchmark suite. Proceedings of the 2012 IEEE Conference on Computer Vision and Pattern Recognition.

[46] Liu C., Gu S., Van Gool L., Timofte R. Deep Line Encoding for Monocular 3D Object Detection and Depth Prediction. Proceedings of the 32nd British Machine Vision Conference (BMVC 2021).

[47] Chen Y., Liu S., Shen X., Jia J. Dsgn: Deep stereo geometry network for 3d object detection. Proceedings of the IEEE/CVF Conference on Computer Vision and Pattern Recognition.

[48] Zhang Y., Lu J., Zhou J. Objects are different: Flexible monocular 3d object detection. Proceedings of the IEEE/CVF Conference on Computer Vision and Pattern Recognition.

[49] Liu X., Xue N., Wu T. (2021). Learning Auxiliary Monocular Contexts Helps Monocular 3D Object Detection. arXiv.

[50] Weng X., Kitani K. (2019). A baseline for 3d multi-object tracking. arXiv.

[51] Gao A., Pang Y., Nie J., Cao J., Guo Y. (2021). EGFN: Efficient Geometry Feature Network for Fast Stereo 3D Object Detection. arXiv.

[52] Königshof H., Salscheider N.O., Stiller C. Realtime 3d object detection for automated driving using stereo vision and semantic information. Proceedings of the 2019 IEEE Intelligent Transportation Systems Conference (ITSC).

[53] Ren S., He K., Girshick R., Sun J. (2015). Faster r-cnn: Towards real-time object detection with region proposal networks. arXiv.

[54] Beltrán J., Guindel C., Moreno F.M., Cruzado D., Garcia F., De La Escalera A. Birdnet: A 3d object detection framework from lidar information. Proceedings of the 2018 21st International Conference on Intelligent Transportation Systems (ITSC).

